# Prevalence and Factors Associated With Hazardous Alcohol Consumption Among Motorcycle Taxi Riders in Kinondoni District, Dar-Es-Salaam, Tanzania: A Cross-Sectional Study

**DOI:** 10.24248/EAHRJ-D-19-00008

**Published:** 2019-11-29

**Authors:** Daniel W Kitua, Titus K Kabalimu, Robert R Muindi

**Affiliations:** a Department of Community Medicine, Faculty of Medicine, Hubert Kairuki Memorial University, Dar es Salaam, Tanzania

## Abstract

**Background:**

Hazardous alcohol consumption is a significant public health problem contributing to road traffic accidents in nearly all countries. Despite the fact that motorcycles are involved in more than half of all road traffic accidents in Tanzania, little has been reported about hazardous alcohol consumption among motorcyclists. This study investigated the prevalence and factors associated with hazardous alcohol consumption among motorcycle taxi riders in Kinondoni District, Dar es Salaam.

**Methods:**

A cross-sectional survey was conducted in Kinondoni District in August 2018 among motorcycle taxi riders. Multistage sampling was applied to select the study participants. Data were collected using structured self-administered questionnaires incorporating the Alcohol Use Disorders Identification Test. Analysis was done using IBM SPSS version 20.

**Results:**

A total of 210 individuals participated in the study. Within the study sample, the prevalence of hazardous alcohol consumption was 61.5% (n=128). Hazardous alcohol consumption was positively associated with a positive family history of alcohol consumption (odds ratio [OR] 11.74; 95% confidence interval [CI], 5.14 to 26.79; *P*<.001). Protective factors were younger age (OR 0.09; 95% CI, 0.02 to 0.40; *P*<.001), having a secondary level of education (OR 0.21; 95% CI, 0.04 to 0.99; *P*=.034), having a primary level of education (OR 0.06; 95% CI, 0.01 to 0.26; *P*<.001), and being employed (OR 11.74; 95% CI, 5.14 to 26.79; *P*<.001).

**Conclusion:**

A high prevalence of hazardous alcohol consumption among motorcycle taxi riders was reported along with several associated factors. Interventions to mitigate hazardous alcohol consumption among commercial motor vehicle drivers must be developed and implemented.

## INTRODUCTION

Hazardous drinking is the pattern of alcohol consumption with increased risk of harmful consequences to the consumers and others.^1,2^ On the other hand, harmful drinking refers to alcohol consumption that result into undesired social consequences together with physical and/or mental ill health.^1,3^ However, Hazardous and harmful alcohol consumption are used as a corresponding terms by the World Health Organization (WHO) referring to patterns of alcohol consumption that are of public health significance despite the absence of any concurrent disorder in the individual user.^4,5^

Hazardous alcohol consumption poses a significant public health and safety problem in nearly all countries, mostly affecting the adolescents and young adults.^6^ Surpassing HIV/AIDS, violence, and tuberculosis, 5.9% of all deaths worldwide are attributed to harmful alcohol consumption.^6,7^ Excessive alcohol consumption is estimated to cause over 10% of the burden of noncommunicable diseases globally including cancers, cerebrovascular and cardiovascular events.^7,8^ Moreover, it is also a contributing factor to communicable diseases such as HIV/AIDS and Tuberculosis.^9,10^ In 2010, the global prevalence of hazardous alcohol consumption was estimated to be 4.1%; whereas Africa region had a prevalence of 3.3% compared to 2.2% and 0.3% of South-East Asia Region and Latin Eastern Mediterranean Region respectively.^7^ In Tanzania, the prevalence of hazardous alcohol consumption was 5.5% with male predominance at 9.3% compared to 1.9% in females as per 2010.^11^ In the findings of a cross-sectional study conducted in Dar-es-Salaam, the prevalence of hazardous alcohol consumption was reported to be 5.7%.^12^

Studies conducted in developing countries have established several factors that are positively associated to hazardous alcohol consumption which include male gender, older age, economic inactivity, high disposable income, high number of lifetime sexual partners and psychological distress.^12–14^ In developed countries, factors associated with increased risk of hazardous alcohol consumption included substance use (cocaine or marijuana, for example) and working in cities with a higher marginalisation index.^15,16^

Within the past 2 decades, road traffic accidents in Africa have escalated from 40.7 to 92.9 per 100,000, and yet alcohol consumption remains the main contributing factor.^17–19^ To our knowledge, there is limited information on the magnitude of hazardous alcohol consumption among motorcycle taxi riders provided that 21.7% of all road traffic accidents in Tanzania are suspected to be under the influence of alcohol and 53.4% of all road traffic accidents involved motorcycles.^20^ In the current study, we assessed the prevalence and factors associated with hazardous alcohol consumption among motorcycle taxi riders in Kinondoni District, Dar es Salaam. Determining the prevalence and factors associated with hazardous alcohol consumption will be essential in raising awareness on the magnitude of the problem and guiding the development of necessary interventions aimed at reduction of all types of alcohol-related harm including road traffic accidents.

## METHODS

### Study Area, Period, and Design

**FIGURE 1.**
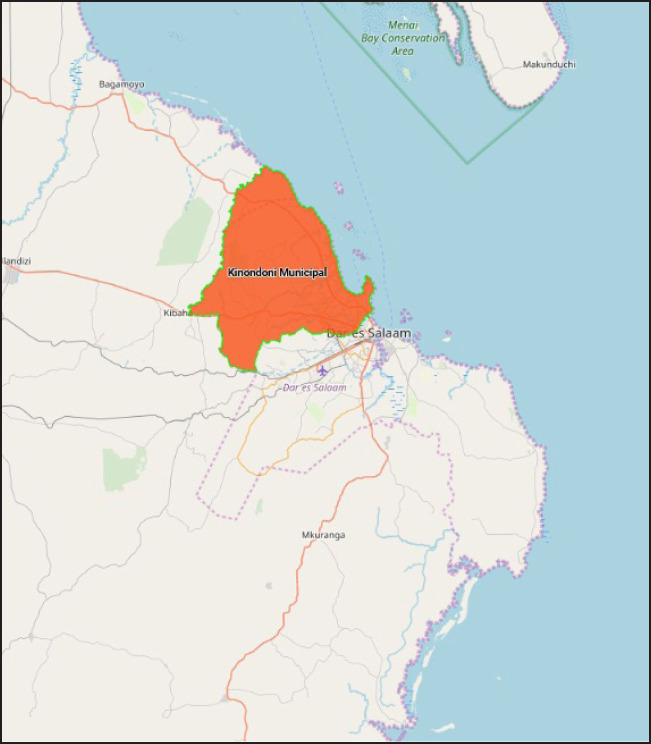
Study Location

A cross-sectional survey was conducted in August 2018 in Kinondoni District, 1 of the 5 districts of Dar es Salaam; others being Temeke, Ilala, Ubungo and Kigamboni (Figure 1). Due to rapid urbanisation, Dar es Salaam inhabits people from diverse ethnic backgrounds, whereas motorcycle taxi has been a rising and common mode of public transport in the city. With a population size of more than 4.3 million people, Dar es Salaam had 302,169 registered motorcycles by May 2016.^18,19^ According to the National Police data, out of 776 road traffic accidents that occurred in Tanzania between January and March 2019, 416 (53%) occurred in Dar es Salaam. For the purpose of this survey, Kinondoni District was selected in order to represent a socioeconomic diverse community in order to ensure maximum variation within the primary data. The population of Kinondoni District in the year 2012 was 1.7 million, comprising over three-quarters of the Dar es Salaam's population as per 2012 general census.^21^ Administratively, Kinondoni Municipal has 4 divisions namely Magomeni, Kinondoni, Kibamba and Kawe. These divisions are divided into 34 wards which are subsequently divided into 171 subwards.

### Sample Size Estimation

The prevalence (10.3%) of hazardous/harmful alcohol consumption among casual laborers in Tanzania was used as the proportion of the problem of interest within population.^12^ Cochrane formula for cross-sectional studies was used to calculate the minimum required sample size as stated below. Cochrane formula:

$$\text{SS}=\frac{Z^{2}\times \text{P}(1-\text{P})}{e^{2}}$$

Where:

SS=minimum desired sample size.

Z=1.96 standard normal deviation at a 95% confidence interval (CI).

P=0.103 (10.3%) proportion of the problem of interest within population.

e=0.05 (5%) which is the margin of standard error.

Substituting these values in the formula gave an approximated minimum required sample size of 142.

### Sampling and Selection of Participants

**FIGURE 2.**
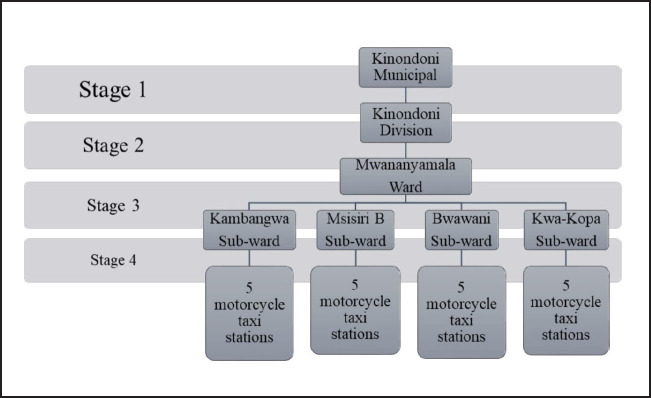
Staged Sampling Procedure

A total of 20 motorcycle taxi stations (waiting areas) were selected, 5 from each of the selected subwards within Mwanayamala administrative ward. The selection applied a multistage cluster sampling technique (Figure 2) as stated below.

Stage 1: The 4 divisions of Kinondoni Municipality (Magomeni, Kinondoni, Kibamba and Kawe) were grouped as clusters and 1 division (Kinondoni) was selected using lottery technique.

Stage 2: All wards within the selected division were identified and grouped as clusters and Mwanayamala administrative ward was selected using lottery technique.

Stage 3: Subwards within Mwanayamala administrative ward were identified and grouped as clusters and 4 subwards (Kambangwa, Msisiri B, Bwawani and Kwa Kopa) were selected using lottery technique.

Stage 4: Motorcycle taxi stations within each selected subwards were identified and grouped as clusters. Simple random sampling technique was applied to select 5 stations from each selected subward in order to obtain a minimum of 142 participants.

Inclusion criteria in the study were: being a duty-bond motorcycle taxi rider with a motorcycle that is registered by the competent agencies, stationed at the selected motorcycle taxi stations and having signed the informed consent form. Exclusion criteria was: cyclists not stationed at the selected taxi stations.

### Assessment of Hazardous Alcohol Consumption

In this study, we employed the Alcohol Use Disorders Identification Test (AUDIT) developed by WHO to aid the identification of individuals with hazardous and harmful patterns of alcohol consumption.^1^

The AUDIT is a scoring system with scores ranging from zero to forty obtained from 10 systematic set questions. In this study, a total score of 8 and above was considered hazardous alcohol consumption.

### Data Collection

After explaining to the participants about the study and its purposes, a self-administered structured questionnaire incorporating the AUDIT (Self-Report Version) was used to collect information on the participants’ social-demographic characteristics and information on the study variables. The questionnaires were translated into Swahili language to aid data collection for participants not conversant with English. It was explained to the participants what was meant by ‘alcoholic drinks’ by using examples of common available alcoholic beverages in a printed pictorial display embodying an alcohol unit reference. Pretesting of the questionnaire in order to ascertain the areas that needed modification was conducted among 20 motorcycle taxi riders in 1 subward (Msisiri A) within the study area by a trained personnel selected from the target population.

### Data Analysis

The collected data were entered and analysed using SPSS version 20 (IBM Corp., Armonk, NY, USA). Descriptive analysis was done using frequencies and proportions. The Dependent variable was hazardous alcohol consumption (AUDIT score ≥8). A score of less than 8 was considered to be low-risk, nonhazardous alcohol consumption; a score between 8 and 15 was considered to be increasing risk; a score between 16 and 19 was considered to be high-risk and a score of 20 and above was considered possible alcohol dependence.

Independent variables were age, gender, marital status, education level, employment status, substance use, family history of alcohol consumption, type of alcoholic beverage consumed and source of alcoholic beverage. Prevalence of hazardous alcohol consumption was expressed as percentage for the entire study population. Chi-square test was used to calculate the association between the dependent and independent variables. Fisher's exact was considered for frequencies less than 5 in any cell or a total frequency less than 20 from all the cells in the contingency table. For each independent variable, the category with the least proportion of hazardous alcohol consumers was used as a reference group and odds ratios (ORs) with 95% CIs. *P*<.05 was considered statistically significant.

### Ethical Considerations

Ethical clearance to conduct the study (Ref: HK/ERC/60/11) was requested and obtained from the Hubert Kairuki Memorial University's Institutional Research Ethics Committee and the Kinondoni Municipal Council was requested by the University to give permit to perform the study in the District. Informed consent was sought after explaining the purpose of the study. Participants who met the inclusion criteria and who were voluntarily willing to participate in the study were enrolled. Permission to withdraw from the study at any time was granted. The identity of the study participants and the motorcycles were made anonymous. Participant were not required to give their names or the motorcycle registration number. This anonymity was explained to participants in the consent form provided to them. Among the 210 contacted individuals meeting the inclusion criteria, all accepted to participate and none of them withdrew from study.

## RESULTS

The minimum required sample size was 142; we manage to contact and enrol 210 participants in the study, thus giving an overall response rate of 100%. Table 1 shows that among 210 participants, 18 (8.6%) were aged below 18 years. Almost half (n=98, 47.1%) of the participants were married and 8 (3.8%) were widowed. About half (n=110, 52.4%) of the participants had a secondary school level of education while 10 (4.8%) had no formal education. About two-thirds (n=136, 66.0%) of the participants were employed, while 26 (12.6%) were unemployed and 44 (21.4%) were self-employed.

**TABLE 1.** Demographic Characteristics

Variable	n	%
**Age, years**		
<18	18	8.6
18–29	90	42.9
30–39	84	40.0
>39	18	8.6
Total	210	100.0
**Sex**		
Male	210	100.0
Female	0	0.0
Total	210	100.0
**Marital status**		
Single	50	24.0
Married	98	47.1
Separated	20	9.6
Divorced	32	15.4
Widowed	8	3.8
Total	208	100.0
**Education level**		
None	10	4.8
Primary	62	29.5
Secondary	110	52.4
Post-secondary	28	13.3
Total	210	100.0
**Employment status**		
Unemployed^a^	26	12.6
Employed^b^	136	66.0
Self-employed^c^	44	21.4
Total	206	100.0

a Using someone's bike and not under contract

b Working under contract

c cOwning a motorcycle taxi

### Prevalence of Hazardous Alcohol Consumption

Within the study population, the prevalence of hazardous alcohol consumption (AUDIT score ≥8) was 61.5% (n=128). Table 2 shows that among 208 participants, 80 (38.5%) were nonhazardous alcohol consumers (AUDIT score <8), 36 (17.3%) had increasing risk, about one-quarter (n=50, 24.0%) were high-risk alcohol drinkers, while 42 (20.2%) had possible alcohol dependence.

**TABLE 2.** Prevalence of Hazardous Alcohol Use by AUDIT Cut-Off Scores

Variable	n	%
0–7 (low-risk/nonhazardous)	80	38.5
8–15 (increasing risk)	36	17.3
16–19 (higher risk)	50	24.0
20 or above (possible dependence)	42	20.2

Abbreviation: AUDIT, Alcohol Use Identification Test

### Factors Associated With Hazardous Alcohol Use

Table 3 shows that young age 18-29 years (OR 0.09; 95% CI, 0.02 to 0.40; *P*<.001), secondary school level of education (OR 0.21; 95% CI, 0.04 to 0.99; *P*<.034), primary school level of education (OR 0.06; 95% CI, 0.01 to 0.26; *P*<.001) and being employed (OR 0.14; 95% CI, 0.06 to 0.35; *P*<.001) were negatively associated with hazardous alcohol consumption. On the other hand, respondents who reported to have a positive family history of alcohol consumption were 11.74 times more likely to be hazardous drinkers compared with those who reported to have no family history of alcohol consumption (OR 11.74; 95% CI, 5.14 to 26.79; *P*<.001). Other factors, such as marital status, substance use, type of alcoholic beverage consumed and source of alcoholic beverage were not significantly associated (*P*>.05) with hazardous alcohol consumption.

**TABLE 3.** Factors Associated With Hazardous Alcohol Consumption

Variables	Nonhazardous alcohol users	Hazardous alcohol users	OR (95% CI)	*P* Value
**Age (years)**				
<18	0	18	–	.486
18–29	52	36	0.09 (0.02–0.40)	<.001
30–39	26	58	0.28 (0.06–1.30)	.143
>39	2	16	1	
**Sex**				
Male	80	128	–	
Female	0	0	–	
**Marital status**				
Single	12	36	1.00 (0.18–5.63)	1.00
Married	54	44	0.27 (0.05–1.41)	.145
Separated	6	14	0.78 (0.12–5.02)	1.00
Divorced	6	26	1.44 (0.23–9.00)	.650
Widowed	2	6	1	
**Education level**				
None	0	10	–	1.00
Primary	16	44	0.21 (0.04–0.99)	.034
Secondary	62	48	0.06 (0.01–0.26)	<.001
Post-secondary	2	26	1	
**Employment status**				
Unemployed	0	24	–	.083
Employed	72	64	0.14 (0.06–0.35)	.000
Self-employed	6	38	1	
**Family history of alcohol use**				
No	72	46	1	
Yes	8	60	11.74 (5.14–26.79)	.000
Do not know	0	22	–	
**Substance use**				
No	70	0	–	
Alcohol only	8	76	0.37 (0.07–1.79)	.315
Alcohol and cigarette	2	52	1	
**Type of alcoholic beverages consumed**				
Beer	5	86	4.3 (0.72–25.83)	.142
Wine	2	8	1	
Whisky	0	14	–	.163
Traditional alcohol	0	2	–	.521
Beer and wine	0	2	–	.521
Beer and traditional alcohol	0	2	–	.521
**Source of alcoholic beverages**				
Shop	4	32	1	
Bar	2	76	4.75 (0.83–27.27)	.078
Shop and bar	0	2	–	1.00
Other	0	8	–	1.00

Abbreviations: OR, odds ratio; CI, confidence interval

## DISCUSSION

This study was able to determine the prevalence and factors associated with hazardous alcohol consumption among motorcycle taxi riders in Kinondoni District, Dar es Salaam. The study determined that a significant proportion (61.5%) of motorcycle taxi riders were hazardous alcohol consumers. Hazardous alcohol consumption was significantly associated with age, level of education, employment status and family history of alcohol consumption.

The prevalence (61.5%) in our study was greater than the estimates in previous studies conducted in sub-Saharan Africa including Tanzania, Ethiopia and Kenya.^12,13,14,22^ The observed difference could be attributed by the dissimilarity of the target populations between the studies. We also found that the prevalence in this study was in agreement with a previous study conducted in the United States among adults and 1 conducted in Ireland among university students.^23,24^

This study also identified several factors associated with hazardous alcohol consumption. Participants aged 18-29 years were less likely to be hazardous alcohol drinkers compared to those aged 39 years and over. Social norms and parental restrictions on alcohol consumption among young people could explain this difference.^13^ This association has been also been demonstrated by Teferra, et al^14^ showing a positive association between old age and hazardous alcohol consumption.

A study conducted by Devaux and Sassi 2015, found that more-educated women were more likely to engage in hazardous alcohol consumption as compared to less-educated women; while, less-educated men were more likely than more-educated men to engage in hazardous drinking.^25^ However, in our study that constituted men only, having a primary or secondary school level of education was negatively associated with hazardous alcohol consumption as compared to participants with post-secondary school level of education. These findings can be attributed to the fact that higher levels of education are linked to high disposable income resulting in higher rates of alcohol consumption as an indulgence.

Employed participants were less likely to be hazardous alcohol consumers as compared to self-employed participants. This could be explained by the contract regulations and restricting that limit the employee's freedom of alcohol consumption. Moreover, the employee has as an obligation to submit/pay part of the income to the employer periodically, thus reducing the disposable income which is a factor that has been linked to hazardous alcohol consumption.^12^

Positive family history of alcohol abuse and low socioeconomic status have a positive association with increased risk of problematic drinking.^26,27^ In this study, a history of alcohol consumption among family members was also found to be positively associated to hazardous alcohol consumption. Together with socioeconomic behaviour of the family, genetic predisposition also influences the likelihood of developing alcohol use disorders among family members.^28,29^

### Limitations

This study had some limitations that need to be pointed out. The cross-sectional study design that was employed could not demonstrate the temporal relationship between the dependent and independent variables. The design was also unable to establish qualitative data that could be essential in augmenting the quantitative findings of the study. Although a good number of risk factors associated with hazardous alcohol consumption was assessed, some factors such as gender, mental disorders and economic status were not assessed. The results relied on reported data that were retrospectively recalled, thus inaccuracies may have been due to recall bias. Data were also collected using self-administered questionnaires; this subjected the study to reporting bias, which could result into overestimating or underestimating the effects of association and the proportion of the findings.

## CONCLUSION AND RECOMMENDATIONS

This study was able to demonstrate that a significant proportion of motorcycle taxi riders in Kinondoni District were hazardous alcohol consumers with possible alcohol dependence. A positive family history of alcohol consumption was an identified factor that was positively related to hazardous drinking among the study participants, thus public health interventions should be address this determinant. In addition to that, more research on this subject matter need to be conducted in order to explore other factors related to hazardous alcohol consumption that were not assessed by the current study and determine the direction of causal linkages.

## References

[1] Babor T, Higgins-Biddle J, Saunders J, Monteiro M. Alcohol Use Disorders Identification Test Guidelines For Use In Primary Care. 2nd ed. Geneva: World Health Organization; 2001.

[2] National Collaborating Centre for Mental Health. Alcohol-Use Disorders: Diagnosis, Assessment And Management Of Harmful Drinking And Alcohol Dependence. London: The British Psychological Society and The Royal College of Psychiatrists; 2011. 22624177

[3] Reid MC, Fiellin DA, O'Connor PG. Hazardous and harmful alcohol consumption in primary care. Arch Intern Med. 1999;159(15):1681–1689. 10.1001/10.1001/archinte.159.15.1681. Medline 10448769

[4] World Health Organization (WHO). Abuse (drug, alcohol, chemical, substance or psychoactive substance). WHO Website. http://www.who.int/substance_abuse/terminology/abuse/en/.

[5] World Health Organization (WHO). Lexicon of alcohol and drug terms published by the World Health Organization. WHO Website. http://www.who.int/substance_abuse/terminology/who_lexicon/en/.

[6] World Health Organization (WHO). Global Status Report on Alcohol and Health. Geneva: WHO; 2011.

[7] World Health Organization (WHO). Global Status Report on Alcohol and Health. Geneva: WHO; 2014.

[8] World Health Organization (WHO). Harmful use of alcohol. WHO Regional Office for the Eastern Mediterranean Website. http://www.emro.who.int/noncommunicable-diseases/causes/harmful-use-of-alcohol.html.

[9] Rehm J, Shield KD, Joharchi N, Shuper PA. Alcohol consumption and the intention to engage in unprotected sex: systematic review and meta-analysis of experimental studies. Addiction. 2012;107(1):51–59. 10.1001/10.1111/j.1360-0443.2011.03621.x. Medline 22151318

[10] Imtiaz S, Shield KD, Roerecke M, Samokhvalov AV, Lönnroth K, Rehm J. Alcohol consumption as a risk factor for tuberculosis: meta-analyses and burden of disease. Eur Respir J. 2017;50(1). 10.1001/10.1183/13993003.00216-2017. Medline PMC5540679 28705945

[11] World Health Organization (WHO). United Republic of Tanzania – alcohol consumption: levels and patterns. WHO Website. http://www.who.int/substance_abuse/publications/global_alcohol_report/profiles/tza.pdf.

[12] Francis JM, Weiss HA, Mshana G, Baisley K, Grosskurth H, Kapiga SH. The Epidemiology of Alcohol Use and Alcohol Use Disorders among Young People in Northern Tanzania. PLoS One. 2015;10(10):e0140041. 10.1001/10.1371/journal.pone.0140041. Medline 26444441 PMC4596556

[13] Mbatia J, Jenkins R, Singleton N, White B. Prevalence of alcohol consumption and hazardous drinking, tobacco and drug use in urban Tanzania, and their associated risk factors. Int J Environ Res Public Health. 2009;6(7):1991–2006. 10.1001/10.3390/ijerph6071991. Medline 19742167 PMC2738894

[14] Teferra S, Medhin G, Selamu M, Bhana A, Hanlon C, Fekadu A. Hazardous alcohol use and associated factors in a rural Ethiopian district: a cross-sectional community survey. BMC Public Health. 2016;16:218. 10.1001/10.1186/s12889-016-2911-6. Medline 26940221 PMC4778336

[15] Crane HM, McCaul ME, Chander G, et al. Prevalence and factors associated with hazardous alcohol use among persons living with HIV across the US in the current era of antiretroviral treatment. AIDS Behav. 2017;21(7):1914–1925. 10.1001/10.1007/s10461-017-1740-7. Medline 28285434 PMC5628735

[16] Semple SJ, Pitpitan EV, Chavarin CV, et al. Prevalence and Correlates of Hazardous Drinking among female sex workers in 13 Mexican Cities. Alcohol Alcohol. 2016;51(4):450–456. 10.1001/10.1093/alcalc/agv124. Medline 26546017 PMC4922383

[17] Adeloye D, Thompson JY, Akanbi MA, et al. The burden of road traffic crashes, injuries and deaths in Africa: a systematic review and meta-analysis. Bull World Health Organ. 2016;94(7):510–521A. 10.1001/10.2471/BLT.15.163121. Medline 27429490 PMC4933140

[18] World Health Organization (WHO). World Report On Road Traffic Injury Prevention. Geneva: WHO; 2004.

[19] Salum JH, Kitali AE, Bwire H, Sando T, Alluri P. Severity of motorcycle crashes in Dar es Salaam, Tanzania. Traffic Inj Prev. 2019;20(2):189–195. 10.1001/10.1080/15389588.2018.1544706. Medline 30888877

[20] Boniface R, Museru L, Kiloloma O, Munthali V. Factors associated with road traffic injuries in Tanzania. Pan Afr Med J. 2016;23:46. 10.1001/10.11604/pamj.2016.23.46.7487. Medline 27217872 PMC4862800

[21] National Bureau of Statistics (NBS) [Tanzania}. 2012 Population and Housing Census. Dar es Salaam, Tanzania: NBS; 2013.

[22] Luchters S, Geibel S, Syengo M, Lango D, King'ola N, Temmerman M, Chersich MF. Use of AUDIT, and measures of drinking frequency and patterns to detect associations between alcohol and sexual behaviour in male sex workers in Kenya. BMC Public Health. 2011;11:384. 10.1186/1471-2458-11-384. Medline 21609499 PMC3128017

[23] Davoren MP, Shiely F, Byrne M, Perry IJ. Hazardous alcohol consumption among university students in Ireland: a cross-sectional study. BMJ Open. 2015;5(1):e006045. 10.1001/10.1136/bmjopen-2014-006045. Medline PMC4316479 25633284

[24] Wilson SR, Knowles SB, Huang Q, Fink A. The prevalence of harmful and hazardous alcohol consumption in older U.S. adults: data from the 2005-2008 National Health and Nutrition Examination Survey (NHANES). J Gen Intern Med. 2014;29(2):312–319. 10.1001/10.1007/s11606-013-2577-z. Medline 24101531 PMC3912311

[25] Devaux M, Sassi F. Social disparities in hazardous alcohol use: self-report bias may lead to incorrect estimates. Eur J Public Health. 2016;26(1):129–134. 10.1001/10.1093/eurpub/ckv190. Medline 26585784 PMC4851717

[26] Labrie JW, Migliuri S, Kenney SR, Lac A. Family history of alcohol abuse associated with problematic drinking among college students. Addict Behav. 2010;35(7):721–725. 10.1001/10.1016/j.addbeh.2010.03.009. Medline 20359831 PMC3056610

[27] Collins SE. Associations between socioeconomic factors and alcohol outcomes. Alcohol Res. 2016;38(1):83–94. Medline 27159815 10.35946/arcr.v38.1.11 PMC4872618

[28] Edenberg HJ, Foroud T. Genetics and alcoholism. Nat Rev Gastroenterol Hepa tol. 2013;10(8):487–494. 10.1001/10.1038/nrgastro.2013.86. Medline PMC4056340 23712313

[29] Vink JM. Genetics of addiction: future focus on gene × environment interaction?. J Stud Alcohol Drugs. 2016;77(5):684–687. 10.1001/10.15288/jsad.2016.77.684. Medline 27588524

